# Genome-wide transcription landscape of citric acid producing *Aspergillus niger* in response to glucose gradient

**DOI:** 10.3389/fbioe.2023.1282314

**Published:** 2023-10-24

**Authors:** Xiaomei Zheng, Peng Du, Kaiyue Gao, Yimou Du, Timothy C. Cairns, Xiaomeng Ni, Meiling Chen, Wei Zhao, Xinrong Ma, Hongjiang Yang, Ping Zheng, Jibin Sun

**Affiliations:** ^1^ College of Biotechnology, Tianjin University of Science & Technology, Tianjin, China; ^2^ Tianjin Institute of Industrial Biotechnology, Chinese Academy of Sciences, Tianjin, China; ^3^ National Technology Innovation Center of Synthetic Biology, Tianjin, China; ^4^ University of Chinese Academy of Sciences, Beijing, China; ^5^ Chair of Applied and Molecular Microbiology, Institute of Biotechnology, Technische Universität Berlin, Berlin, Germany; ^6^ School of Biotechnology, East China University of Science and Technology, Shanghai, China; ^7^ Shan Dong Fuyang Biological Technology Co., Ltd., Dezhou, China

**Keywords:** *Aspergillus niger*, glucose signaling, CCR, citric acid, carbon utilization

## Abstract

*Aspergillus niger* is the main industrial workhorse for global citric acid production. This fungus has complex sensing and signaling pathways to respond to environmental nutrient fluctuations. As the preferred primary carbon source, glucose also acts as a critical signal to trigger intracellular bioprocesses. Currently, however, there is still a knowledge gap in systems-level understanding of metabolic and cellular responses to this vital carbon source. In this study, we determined genome-wide transcriptional changes of citric acid-producing *Aspergillus niger* in response to external glucose gradient. It demonstrated that external glucose fluctuation led to transcriptional reprogramming of many genes encoding proteins involved in fundamental cellular process, including ribosomal biogenesis, carbon transport and catabolism, glucose sensing and signaling. The major glucose catabolism repressor *creA* maintained a stable expression independent of external glucose, while *creB* and *creD* showed significant downregulation and upregulation by the glucose increase. Notably, several high-affinity glucose transporters encoding genes, including *mstA*, were greatly upregulated when glucose was depleted, while the expression of low-affinity glucose transporter *mstC* was glucose-independent, which showed clear concordance with their protein levels detected by *in situ* fluorescence labeling assay. In addition, we also observed that the citric acid exporter *cexA* was observed to be transcriptionally regulated by glucose availability, which was correlated with extracellular citric acid secretion. These discoveries not only deepen our understanding of the transcriptional regulation of glucose but also shed new light on the adaptive evolutionary mechanism of citric acid production of *A. niger*.

## 1 Introduction

*Aspergillus niger* is an important industrial filamentous fungus, which has been widely exploited for bulk manufacturing of organic acids and enzymes (Meyer et al., 2011; Tong et al., 2019). For instance, *Aspergillus niger* was the major citric acid industrial workhorse for over 80% of worldwide citric acid production, with the global citric acid market of nearly 2.0 million tons (Tong et al., 2019). *Aspergillus niger* has good fitness for industrial production, with powerful hydrolytic enzymes to efficiently convert a wide range of crude raw carbon sources into high-value products (Gruben et al., 2017; Peng et al., 2018). To cope with varying carbon source availability, *A. niger* has evolved a powerful capacity to metabolize a wide range of saccharides, and consequently, complex signaling systems to respond and prioritize various sugars.

Although more complex substrates have been developed as carbon sources for fungal fermentation, monomeric sugars, especially glucose, are curial to understanding the physiology and cognate regulatory pathways that govern growth, development, and productivity in *A. niger*. During fungal growth in both the natural niche and industrial bioprocesses, glucose is the preferred sugar over other carbon sources, which leads to the inactivation of the transport and metabolic pathway involved in the assimilation of alternative carbon sources. This preference was known as carbon catabolite repression (CCR) (Reijngoud et al., 2021), resulting in the repression of catabolizing other alternative carbon sources until glucose was depleted (Makela et al., 2018). In many filamentous fungi, the transcription repressor CreA is considered to be the major component of CCR, and its function and stability are regulated in response to glucose (Reijngoud et al., 2021). Interestingly, however, Mäkelä et al. found that *A. niger* takes up monomeric sugars in a sequential manner which is not mediated by CreA in some instances (Makela et al., 2018). Moreover, the transcription of genes encoding amylolytic enzymes is activated by low carbon availability, in a *creA* mutant of *A. nidulans* (Nakamura et al., 2006). It has also been demonstrated in *Aspergillus nidulans* that deletion of *creA* does not cause significant de-repression of cellulase production in media containing cellulose and glucose (Kunitake et al., 2019). Thus, while CreA is the major component of CCR, it is not the only regulatory component of this process, and further identification of CreA-independent CCR may enable better control of carbon utilization during fermentation. In addition to CCR, the transcription of CAZy genes has been shown to be co-controlled by multiple transcriptional activators, *e.g.*, AmyR and XlnR (Gruben et al., 2017). These regulators also respond to the corresponding saccharides and are activated by these inducers to release the repression of CAZy genes (Gruben et al., 2017) and transporters (Peng et al., 2018), when the preferred sugar, glucose, was depleted. Therefore, as the preferred sugar, glucose not only triggers CCR, but is also crucial in other complicated sugar signaling and regulatory networks.

The glucose sensing and signaling pathway has been well dissected and characterized in baker’s yeast *Saccharomyces cerevisiae* (Brink et al., 2021). Three major sugar signaling networks have been identified: 1) the Snf3/Rgt2 pathway sense external glucose changes and induce the expression of hexose transporters that in turn import glucose into the cells; 2) the Snf1/Mig1 pathway is similar to the CCR in *Aspergilli*, activated by glucose depletion, and regulate the genes involved in the utilization of alternative sugars; 3) the cyclic adenosine monophosphate (cAMP)/protein kinase A (PKA) pathway also involves in the glucose sensing and regulates the metabolism, growth and stress response. In *Aspergilli*, the major signaling pathways include the cAMP/PKA signaling pathway, mitogen-activated protein kinase (MAPK) cascades, and calcium/calcineurin signaling pathway (Cairns et al., 2019). The cAMP/PKA signaling system is considered to be the major glucose sensing and signaling pathway (Zheng et al., 2022b). In *A. nidulans*, the putative GprD carbon receptor induced hyphal growth and conidial germination in the presence of glucose (Han et al., 2004), while the other GprH carbon and amino acid receptor activated glucose uptake during carbon starvation (Brown et al., 2015; Dos Reis et al., 2019). However, no research has determined the transcriptional response of *A. niger* across multiple glucose concentrations, resulting in a major knowledge gap at a systems level to understand the cellular responses to this vital carbon source glucose.

To address this issue, we conducted a transcriptomic analysis of a citric acid-producing isolate *A. niger* D353 under glucose gradient and unveiled a genome-wide transcriptional landscape in response to extracellular glucose availability in *A. niger*. To break the limitation of comparative analysis only between two samples, Fuzzy c-means clustering was performed to depict global co-expression patterns of the differentially expressed genes under multiple glucose levels, which revealed that glucose fluctuation led to transcriptional reprogramming of numerous genes in this industrially important fungus. Thus, the transcriptional responses of the sugar signaling pathway, carbohydrate hydrolysis, and transport, and central metabolism were also investigated in detail. In addition, to functionally verify novel findings gleaned from transcriptional analyses, we also performed an *in situ* fluorescence labeling assay to display the response of glucose transporters to glucose gradient, and, finally, detected citric acid efflux to certify the glucose-dependent regulation of citric acid exporter CexA.

## 2 Materials and methods

### 2.1 Strains and cultivation conditions

The citric acid-producing strain *A. niger* D353 was used to conduct transcriptomic analysis. *Aspergillus niger* D353.8 was derived from D353 with the double deletion of orotidine-5′-phosphate decarboxylase *pyrG* and non-homologous end-joining (NHEJ) component *kusA*, which was used to construct the mCherry labeling mutants because of the *pyrG* selection marker and its elevated precise editing efficiency due to the NHEJ deficiency (Supplementary Table S1) (Zhang et al., 2020). *Aspergillus niger* strains were cultivated on a defined minimal medium (MM) as reported previously, or on a complete medium (CM) consisting of MM supplemented with 0.5% yeast extract and 0.1% casamino acids (Zheng et al., 2022a). For plates, 1.5% agar was supplemented. When necessary, 10 mM uracil was supplemented in the media.

To investigate a genome-wide transcription landscape of *A. niger* responding to glucose gradient, a series of glucose concentrations were selected: no carbon (0%), low glucose (0.2%), intermediate glucose level (2% and 4%), and high glucose level (10%). For pre-culturing, 10^8^ spores of the citric acid producing strain D353 were inoculated into a flask with 100 mL of CM media and incubated for 18 h at 30°C and 200 rpm. After being washed twice in PBS buffer, the mycelia were transferred into the MM media containing different concentrations glucose and incubated for 1 h at 30°C and 200 rpm. After inducing under these glucose concentrations, mycelia were harvested by vacuum-filtration, washed in PBS buffer, and then rapidly frozen in liquid nitrogen. Finally, these mycelia were stored at −80°C for further experiments.

### 2.2 RNA extraction and sequencing

The samples for transcriptomic profiling were prepared according to the previous approach (Zheng et al., 2022b). Total RNAs were extracted using RNAprep pure Plant Kit (DP432, Tiangen, Beijing, China) according to the manufacturer’s instructions. RNA concentration and purity were measured using NanoDrop 2000 (Thermo Fisher Scientific, Wilmington, Germany). RNA integrity was assessed using the RNA Nano 6000 Assay Kit of the Agilent Bioanalyzer 2,100 system (Agilent Technologies, CA, United States). Sequencing libraries were constructed using NEBNext UltraTM RNA Library Prep Kit for Illumina (NEB, United States), and the library preparations were sequenced on an Illumina platform, and paired-end reads were generated at Beijing Biomarker Technology Co., Ltd. (Beijing, China).

A total of 469, 721, 042 clean reads related to 10 cDNA libraries (each condition measured in duplicate) were obtained for assembly and further analysis after filtering and trimming the raw data (Supplementary Table S4). Average clean reads of 42.88, 48.17, 51.40, 48.26, and 44.15 million were generated from the samples under the concentration of 0%, 0.2%, 2%, 4%, and 10% glucose. The percentage of bases with Phred scores at the Q30 level (an error probability of 1‰) ranged from 92.38% to 95.41% and the GC content was 54.18%–54.90%. Clean reads were obtained by removing reads containing adapter, reads containing ploy-N and low-quality reads from the raw reads. These clean reads were then mapped to the reference genome sequence of the progenitor strain which was annotated according to the reference genome of *A. niger* CBS 513.88 (GenBank Accession: PRJNA19263). Among all the samples, 89.15%–91.92% of the clean reads were mapped to the reference genome, in which, 86.64%–90.19% of clean reads were uniquely mapped (Supplementary Table S4). These data have been provided in the Supplementary Materials.

### 2.3 Transcriptomic data analysis

Transcriptomic data were analyzed following the approaches reported in a previous study (Lu et al., 2022). First, the reproducibility of the sample replicates was evaluated by Pearson correlation analysis by Pearson’s Correlation Coefficient (Liu et al., 2018). The principal component analysis (PCA) of the transcriptomic data was visualized on the BMKCloud platform (www.biocloud.net). For gene expression quantification, FPKM (Fragment Per Kilobase of transcript per Million fragments mapped) was applied to measure the expression level of each gene by StringTie using the maximum flow algorithm. Differential expression analysis was performed by edgeR (Robinson et al., 2010). For differentially expressed genes, the fold change (FC) and false discovery rate (FDR) thresholds were set at ≥2 and ≤0.05, respectively. Genes with FPKM value ≥ 10 under any condition were considered to be expressed and selected for further analysis. Venn diagram and hierarchical clustering analysis were performed on the BMKCloud platform, to show the distribution of DGE for the comparisons of glucose gradients. Differentially expressed genes were grouped into different clusters using the Mfuzz R package with a fuzzy c-means algorithm (Kumar and Futschik, 2007). eggNOG orthologous classification of DEGs was performed on the BMKCloud platform using the eggNOG database (http://eggnog5.embl.de/#/app/home) (Cantalapiedra et al., 2021). Fisher’s exact test, which is based on the hypergeometric distribution, was used to calculate the *p*-value, and the eggNOG functional enrichment of DEGs in different clusters with a corrected *p*-value less than 0.05 were considered to be significantly enriched. Then, DEGs clustering function enrichment was visualized by chord diagram using Bioladder (https://www.bioladder.cn/).

### 2.4 Construction of *mCherry*-labeling strains

To construct the *in situ* fluorescence labeling strains, the highly efficient genome editing CRISPR/Cas9 technique was used in this study. All protospacers, primers, and plasmids used in this study are listed in Supplementary Tables S1–S3. To fuse the mCherry at the C-terminal of targets, protospacers were designed using the sgRNAcas9 software (Xie et al., 2014), with minimal off-target possibility, and located at the 5′-uptream of the stop codon. The targeting sgRNA constructs were cloned into the psgRNA6.0 (Zheng et al., 2019), with synthetic double-stranded oligonucleotides of the desired sequences, according to the protocol (Zheng et al., 2022a). The donor DNAs containing 40-bp micro-homology flanks were amplified using pYDD1 (Lu et al., 2022) as a template. The sgRNA, donor DNA, and pCas9-AnpyrG were co-transformed into the protoplasts of *A. niger* D353.8. After twice subcultures and purification, the genomic DNA of randomly selected transformants was extracted and verified via diagnostic PCR and sequencing analysis with the corresponding primers (Supplementary Table S3). Isolates that passed PCR confirmation screens were selected for fluorescence microscopic analysis.

### 2.5 Microscopy and imaging

To confirm the gene expression changes in response to glucose gradients correspond-ing to differences in protein abundance, the hyphae of mCherry labeling strains were pre-pared for the fluorescence microscopic analysis according to a previous study [24]. Briefly, two disinfected coverslips were placed onto the bottom of a small Petri dish containing 5 mL of liquid MM with 0.003% yeast extract. After inoculation with 106 spores for 8 h at 30°C and transferred in the MM with different glucose concentrations, coverslips with ad-herent hyphae were placed upside down on an object slide for the microscope analysis. Differential interference contrast (DIC) and red fluorescent images of the cells were cap-tured with a ×40 objective using a Leica DM5000B laser scanning confocal microscope (Leica, Wetzlar, Germany) with excitation at 543 nm and detection at 586–670 nm. The re-sults were assembled in Adobe Photoshop 7.0 (Adobe, San Jose, CA).

### 2.6 Extracellular citric acid detection

To verify the CexA transcriptional regulation, after pre-cultivated in CM media, the mycelia of D353 were induced in MM media with different glucose concentrations. After induction for 3 h, the supernatants were filtered from cultures using filter paper and were boiled for 15 min at 100°C. Then, the treated supernatants were centrifuged at 12,000 rpm for 5 min and filtered through a 0.22 μm sterile filter membrane. Extracellular citric acid was detected by Prominence UFLC equipped with a UV detector (Shimadzu, Kyoto, Japan) and a Bio-Rad Aminex HPX-87H column (300 × 7.8 mm) according to the procedure de-scribed previously [15].

## 3 Results

### 3.1 Transcriptional response to extracellular glucose availability in *Aspergillus niger*

**FIGURE 1:**
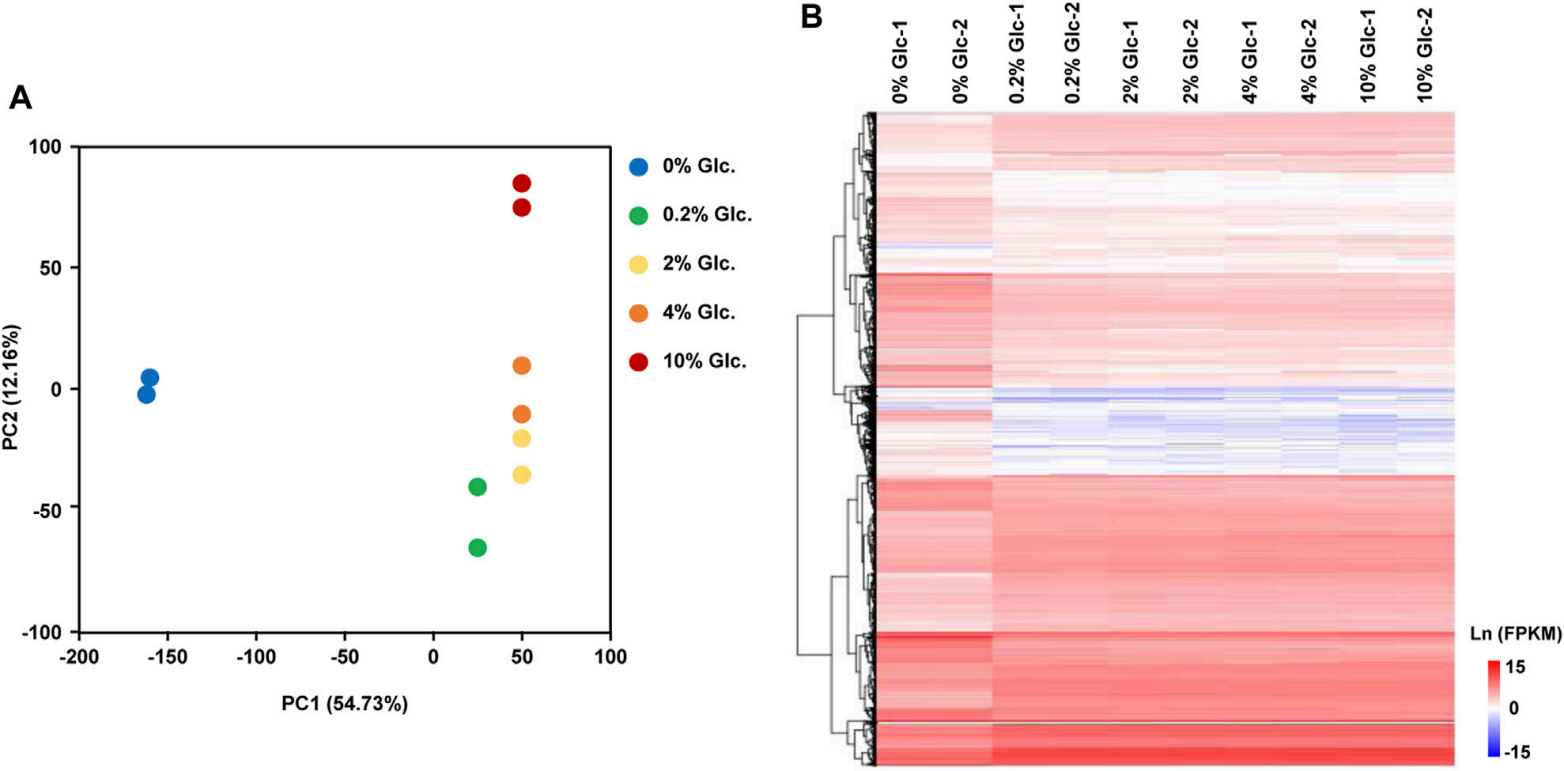
Global transcriptome analysis of *Aspergillus niger* D353 in response to extracellular glucose. **(A)** Principal component analysis (PCA) shows the variance between the individual samples. PC1 and PC2 represented the first principal component and the second principal component of PCA analysis. **(B)** Hierarchical clustering analysis of DEGs in response to glucose gradients. Natural transformed gene expression values (FPKM) were color-coded from blue to red.

To investigate the physiological response to environmental glucose availability, we compared the transcriptomes of mycelia incubated for 1 h under five glucose concentrations (0%, 0.2%, 2%, 4%, and 10%). The transcriptomic data recorded by RNA-sequencing showed a good positive correlation between the sample replicates, with Pearson’s correlation coefficient greater than 0.99 (Supplementary Figure S1). According to the principal component analysis and the hierarchical clustering analysis of differentially expressed genes (Figure 1), glucose had a considerable effect on the transcriptome, especially in the absence of glucose, with PC1 of 54.73%. In *A. niger*, the transcriptional patterns incubated with 0.2%, 2%, and 4% displayed no great difference, while the transcriptional profiling with a glucose concentration of 10% showed clear variation in PC2 (12.16%).

**FIGURE 2:**
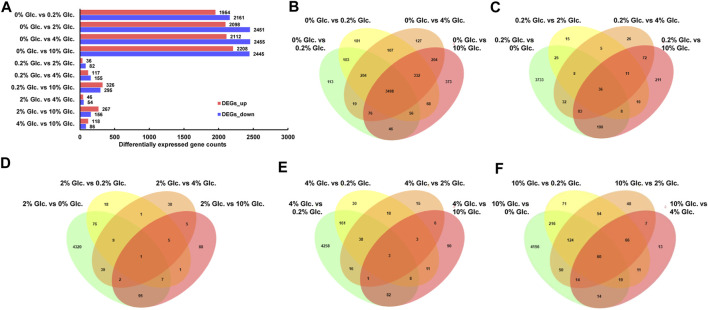
Differentially expressed genes of *Aspergillus niger* in response to external glucose. The number of up-and downregulated genes for the individual comparisons **(A)** and the Venn diagram showing the distribution and overlap of DGE for the comparisons of the different conditions **(B–F)**.

The DGE analysis demonstrated that when comparing pairwise, 5,636 genes were predicted as differentially expressed genes based on the thresholds of FC ≥ 2 and FDR≤0.05 (Figure 2). Among them, in comparison to no glucose, 3,498 genes showed significant expression changes in all other glucose conditions (Figure 2B), suggesting that carbon starvation led to substantial changes in gene expression. Meanwhile, with the rising glucose concentration discrepancy, the number of DEGs gradually increased. For instance, compared with the low glucose level of 0.2%, 49, 57, and 401 specific DEGs were identified under the glucose concentration of 2%, 4%, and 10%, respectively. These data suggested that apart from the considerable impact of carbon starvation, the increase of glucose also caused other transcriptional responses.

**FIGURE 3:**
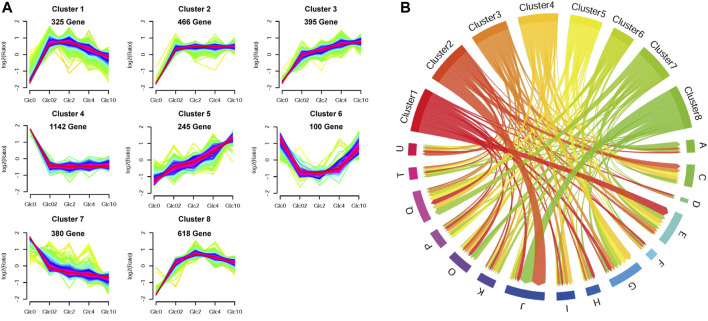
Fuzzy c-means clustering of DEGs **(A)** and chord diagram of eggNOG function enrichment of each expression pattern **(B)**. Fuzzy c-means clustering identified eight distinct gene expression patterns of protein expression. The *x*-axis represents five glucose conditions, while the *y*-axis represents log2-transformed, normalized intensity ratios in each condition. In the chord diagram, the gene functions were represented as A-Z. An RNA processing and modification; B, Chromatin structure and dynamics; C, Energy production and conversion; D, Cell cycle control, cell division, chromosome partitioning; E, Amino acid transport and metabolism; F, Nucleotide transport and metabolism; G, Carbohydrate transport and metabolism; H, Coenzyme transport and metabolism; I, Lipid transport, and metabolism; J, Translation, ribosomal structure and biogenesis; K, Transcription; L, Replication, recombination and repair; M, Cell wall/membrane/envelope biogenesis; N, Cell motility; O, Posttranslational modification, protein turnover, chaperones; P, Inorganic ion transport and metabolism; Q, Secondary metabolites biosynthesis, transport, and catabolism; S, Function unknown; T, Signal transduction mechanisms; U, Intracellular trafficking, secretion, and vesicular transport; V, Defense mechanisms; W, Extracellular structures; Y, Nuclear structure; Z, Cytoskeleton.

To obtain the key genes in response to glucose supply, we clustered the DEGs (FPKM>10) into corresponding profiling patterns by Fuzzy c-means clustering R package (Kumar and Futschik, 2007), based on the changing trends in expression under different glucose conditions. As shown in Figure 3, we obtained 8 clusters with distinct expression patterns. Due to the considerable influence of glucose depletion, genes in Cluster 1, 2, 3, and 8 were mostly repressed in the absence of glucose and were dramatically upregulated when glucose was supplied. The DEGs in these clusters, especially in Cluster 1, Cluster 2, and Cluster 8, were mainly enriched in RNA processing and modification, Translation, ribosomal structure and biogenesis, Amino acid transport and metabolism, and Energy production and conversion. Conversely, genes in Cluster 4, 6, and 7 were activated by glucose depletion, which were involved in Carbohydrate transport and metabolism, Secondary metabolites biosynthesis, transport and catabolism, and Posttranslational modification, protein turnover, and chaperones. In addition, genes in Cluster 5 showed gradually elevated gene expression along with the increase in glucose supply, which was also involved in Carbohydrate transport and metabolism.

### 3.2 Effect of glucose gradients on the glucose signaling pathway

To investigate the effect of glucose on cell signaling pathways, we performed the detailed transcription profiling of signaling cascades in response to external glucose in *A. niger* (Table 1). The cAMP/PKA signaling pathway was most influenced by the external glucose changes, especially the G-protein coupled receptors (GPRCs), G-proteins, and their regulators (Table 1). Different from the finding of GPRCs in *A. nidulans* (Brown et al., 2015; Dos Reis et al., 2019), there are no detectable transcriptional changes of the orthologs of the major receptors GprD (An02g01560) and GprH (An02g01560) in *A. niger*. In contrast, GprM (An07g02930) and GprF (An08g04110), with the transcription pattern of Cluster 7, were significantly upregulated up to 2.46-fold and 2.64-fold under carbon starvation, compared with the condition of 10% glucose. In contrast, the other two GPRCs GprO (An16g04540) and GprJ (An18g05870), belonged to Cluster 8, showed reverse expression trends, which were upregulated by glucose, up to 2.56-fold and 2.05-fold at the glucose level of 4%. Similarly, the G-protein α-subunits also displayed different transcription patterns. FadA remained stable expression under varying glucose concentrations, while the other two G-protein α-subunits GanA and GanB were significantly regulated by glucose. GanA was upregulated up to 3.34-fold by 2% glucose, but GanB showed the opposite pattern and was induced about 1.87-fold when glucose was depleted. In addition, G-protein beta subunit SfaD and Gβ-like protein CpcB were upregulated by glucose, about 1.27-fold and 7.30-fold at 0.2% glucose compared with no glucose, which showed a similar transcription pattern of GanA. The regulator of GanB signaling, RgsA (An18g06110), and PKA regulatory subunit PkaR (An16g03740) were also activated by the increase of external glucose. These data implied that there were two distinct glucose sensing and signaling pathways to respond to carbon starvation and glucose usability, respectively.

**TABLE 1:** Differentially expressed genes involved in cell signaling in response to external glucose change.

Gene ID	Cell signaling	Gene name	Fuzzy Cluster^a^	0% Glc.^b^	0.2% Glc.^b^	2% Glc.^b^	4% Glc.^b^	10% Glc.^b^
An02g01560	cAMP/PKA	*gprD*	*n.d.*	5.85 ± 0.01	5.65 ± 0.31	5.46 ± 0.18	5.32 ± 0.79	6.74 ± 0.58
An13g01340	cAMP/PKA	*gprH*	*n.d.*	12.3 ± 0.18	22.1 ± 2.22	21.27 ± 0.84	20.62 ± 0.97	19.36 ± 0.25
An07g02930	cAMP/PKA	*gprM*	7	65.01 ± 2.86	40.05 ± 1.52	31.51 ± 0.94	26.32 ± 0.23	26.41 ± 0.26
An08g04110	cAMP/PKA	*gprF*	7	44.34 ± 1.18	24.05 ± 0.17	18.25 ± 0.06	19.75 ± 0.15	16.77 ± 0.56
An16g04540	cAMP/PKA	*gprO*	8	26.68 ± 1.65	51.45 ± 1.23	61.12 ± 0.33	65.55 ± 1.84	56.74 ± 0.9
An18g05870	cAMP/PKA	*gprJ*	8	9.68 ± 0.79	16.77 ± 0.38	17.67 ± 2.25	19.91 ± 0.86	18.85 ± 0.21
An08g06130	cAMP/PKA	*fadA*	*n.d.*	173.94 ± 1.52	182.25 ± 12.98	188.6 ± 13.12	192.79 ± 12.65	179.02 ± 5.84
An02g08000	cAMP/PKA	*ganA*	3	82.88 ± 0.92	212.25 ± 2.24	276.49 ± 4.5	254.2 ± 4.23	279.01 ± 10.79
An08g05820	cAMP/PKA	*ganB*	7	87.02 ± 0.78	48.49 ± 3.75	46.51 ± 2.28	49.12 ± 0.51	53.05 ± 0.49
An18g02090	cAMP/PKA	*sfaD*	3	87.61 ± 2.72	105.47 ± 4.69	111.59 ± 5.55	108.31 ± 7.13	105.76 ± 5.06
An01g08850	cAMP/PKA	*cpcB*	8	148.69 ± 5.67	903.3 ± 38.99	1,085.85 ± 34.58	995.29 ± 17.51	892.67 ± 63.61
An18g06110	cAMP/PKA	*rgsA*	2	46.37 ± 0.62	95.75 ± 4.15	105 ± 0.61	95.9 ± 2.16	103.73 ± 7.75
An16g03740	cAMP/PKA	*pkaR*	3	64.03 ± 2.32	102.79 ± 4.1	85.37 ± 2.59	94.01 ± 0.33	113.27 ± 7.36
An03g06820	MAPK	*regA*	4	52.28 ± 1.62	24.66 ± 0.58	22.57 ± 0.03	20.2 ± 0.10	21.44 ± 0.89
An08g10670	MAPK	*mpkB*	4	164.81 ± 3.69	90.51 ± 2.37	88.75 ± 1.61	82.94 ± 2.04	76.83 ± 1.67
An07g03980	MAPK	*hogA*	7	68.40 ± 2.02	25.35 ± 2.32	23.11 ± 0.97	24.57 ± 1.39	17.43 ± 0.48
An08g05850	MAPK	*mpkC*	6	173.96 ± 4.81	122.9 ± 3.67	130.79 ± 7.12	147.59 ± 2.64	167.91 ± 2.28
An11g03170	MAPK	*HP1*	8	34.47 ± 0.21	124.79 ± 7.41	157.44 ± 4.42	155.84 ± 2.61	134.52 ± 0.58
An04g07010	CaM	*caM*	2	240.33 ± 12.4	514.12 ± 46.8	496.09 ± 37.74	457.97 ± 40.27	494.36 ± 5.71

^a^ Note: Fuzzy Cluster corresponded to the transcriptional patterns analyzed by Fuzzy c-means clustering of DEGs. The *n.d*. represented that the genes were not identified as DEGs, and had no corresponding transcriptional pattern clusters.

^b^ The expression value of each gene under different glucose concentrations was shown as FPKM, value with their standard deviation.

In addition to cAMP/PKA signaling, some major components in the MAPK signaling cascade and calcium/calcineurin signaling pathway were also regulated at the transcription level (Table 1). For instance, a histidine-containing phospho-transmitter (HP, An11g03170), and calmodulin CaM (An04g07010) were upregulated by the glucose increase. In contrast, RegA (An03g06820) and MpkB (An08g10670) in the nutrient-sensing MAPK signaling, and HogA (An07g03980) in the High Osmolarity Glycerol pathway (HOG) were significantly activated by the glucose depletion. Meanwhile, MpkC (An08g05850) in the HOG signaling pathway showed higher expression both at the condition of glucose depletion and high glucose level, suggesting this gene might be involved in the complicated regulation of carbon starvation and high glucose osmolarity. Taken together, these data implied that *A. niger* evolved complex glucose sensing and signaling pathway to cope with external glucose fluctuation.

### 3.3 Effect of glucose gradients on carbon utilization and regulation

As the preferred sugar, glucose triggers CCR, which acts as a signal to induce genes involved in glycolysis and simultaneously repress genes in the metabolic pathways of other alternative carbon sources. To unveil the full view of the response of carbohydrate utilization to glucose, we analyzed the DEGs involved in various carbon utilization and regulation. First, CreA was the major repressor in CCR regulation, but it was unexpected that CreA maintained the stable expression at all the tested conditions in a glucose-independent way (Table 2). Similarly, SnfA and some factors interacting with CreA, including putative co-repressors RcoA and SsnF, protein kinase CkiA, and ubiquitin protein degradation associated Fbx23, displayed no detectable changes under all tested glucose level. By contrast, a glycogen synthase kinase GskA, which might be involved in the phosphorylation regulation of CreA, was considerably induced by the glucose supply. Meanwhile, the other two factors involved in CCR, CreB, and CreD were significantly influenced by external glucose. The deubiquitinating enzyme CreB was induced by carbon starvation and repressed in the presence of glucose, while arrestin-related trafficking adaptor (ART) protein CreD showed the opposite expression pattern, dramatically upregulated when glucose was available, up to 6.19-fold and 7.91-fold at the glucose concentration of 0.2% and 2% (Table 2). These data implied that the CreA-mediated CCR responding glucose might not be dependent on the transcription of CreA.

**TABLE 2:** Differentially expressed carbohydrate utilization regulators in response to external glucose change.

Substrate	Gene ID	Gene name	Fuzzy Cluster^a^	0% Glc.^b^	0.2% Glc.^b^	2% Glc.^b^	4% Glc.^b^	10% Glc.^b^
various	An02g03830	*creA*	*n.d.*	121.44 ± 5.67	147.77 ± 5.52	128.25 ± 15.52	132.67 ± 9.94	128.84 ± 4.92
various	An01g08470	*creB*	4	77.69 ± 1.40	29.61 ± 4.82	27.16 ± 4.64	26.43 ± 1.65	23.34 ± 1.28
various	An11g02750	*creC*	*n.d.*	68.6 ± 1.57	79.15 ± 1.83	71.8 ± 1.98	80.22 ± 0.99	80.52 ± 3.44
various	An11g02830	*creD*	2	64.38 ± 2.44	398.75 ± 57.17	509.38 ± 58	487.58 ± 55.12	488.55 ± 4.26
various	An03g04720	*snfA*	*n.d.*	35.88 ± 0.52	36.72 ± 0.43	39.94 ± 3.65	43.82 ± 3.44	40.81 ± 2.41
various	An15g00140	*rcoA*	*n.d.*	128.79 ± 3.00	137.71 ± 3.76	145.13 ± 1.36	144.03 ± 3.05	129.23 ± 1.16
various	An02g03940	*ssnF*	*n.d.*	60.18 ± 1.84	62.65 ± 5.65	67.63 ± 5.94	62 ± 4.45	56.29 ± 0.84
various	An07g06080	*ckiA*	*n.d.*	510.92 ± 4.65	520.67 ± 15.22	534.73 ± 23.33	534.61 ± 9.45	516.05 ± 8.13
various	An15g01700	*gskA*	2	198.68 ± 4.85	642.57 ± 7.56	696.53 ± 0.67	684.49 ± 3.77	605.42 ± 1.88
various	An04g05180	*fbx23*	*n.d.*	35.42 ± 0.84	41.61 ± 4.01	48.75 ± 6.11	55.01 ± 6.58	50.82 ± 0.70
starch	An04g06910	*amyR*	7	129.15 ± 2.46	66.26 ± 20.1	48.66 ± 6.77	46.53 ± 2.95	48.51 ± 1.2
cellulose	An05g00020	*clrA*	4	32.18 ± 0.18	9.74 ± 1.13	7.88 ± 0.73	8.05 ± 0.49	7.79 ± 0.23
various	An12g01870	*clrB*	7	188.43 ± 0.31	72.32 ± 7.13	66.77 ± 1.09	73.54 ± 2.43	79.1 ± 0.89
various	An15g05810	*xlnR*	4	73.88 ± 1.41	14.78 ± 2.01	11.1 ± 0.83	12.03 ± 0.15	12.76 ± 0.43
pectin	An04g00780	*gaaR*	4	67.73 ± 0.13	9.78 ± 1.04	6.3 ± 0.21	6.06 ± 0.39	7.99 ± 0.26
pectin	An04g00790	*gaaX*	4	52.87 ± 0.01	12.67 ± 1.68	12.67 ± 0.19	10.93 ± 0.25	10.63 ± 1.08
pectin	An13g00910	*rhaR*	4	16.46 ± 0.71	2.64 ± 3.08	2.05 ± 0.12	2.87 ± 0.49	2.48 ± 0.14

^a^ Note: Fuzzy Cluster corresponded to the transcriptional patterns analyzed by Fuzzy c-means clustering of DEGs. The *n.d*. represented that the genes were not identified as DEGs, and had no corresponding transcriptional pattern clusters.

^b^ The expression value of each gene under different glucose concentrations was shown as FPKM, value with their standard deviation.

As to carbon utilization, the transcriptions of 7 key transcription factors and 52 genes encoding Carbohydrate-Active enzymes (CAZy) were significantly regulated by external glucose (Table 2; Supplementary Table S5). Among these DEGs, 46 genes (77.96%, 46/59), displayed the expression pattern of Cluster 4, which indicated that these genes were rigorously inhibited by CCR to inhibit the utilization of alternative carbon sources. The related transcription factors, including cellulose activators ClrA, xylan activators XlnR, and pectin utilization regulators GaaR, GaaX, and RhaR, were only significantly induced at carbon starvation and repressed by glucose. By comparison, amylolytic regulator AmyR, the major glucoamylase GlaA and cellulose activator ClrB, were also downregulated by glucose but remained certain of expression at high glucose levels (Table 2). These data were also consistent with the CCR regulation that when glucose was present, the polysaccharide hydrolysis was repressed.

### 3.4 Effect of glucose gradients on sugar transport

**FIGURE 4:**
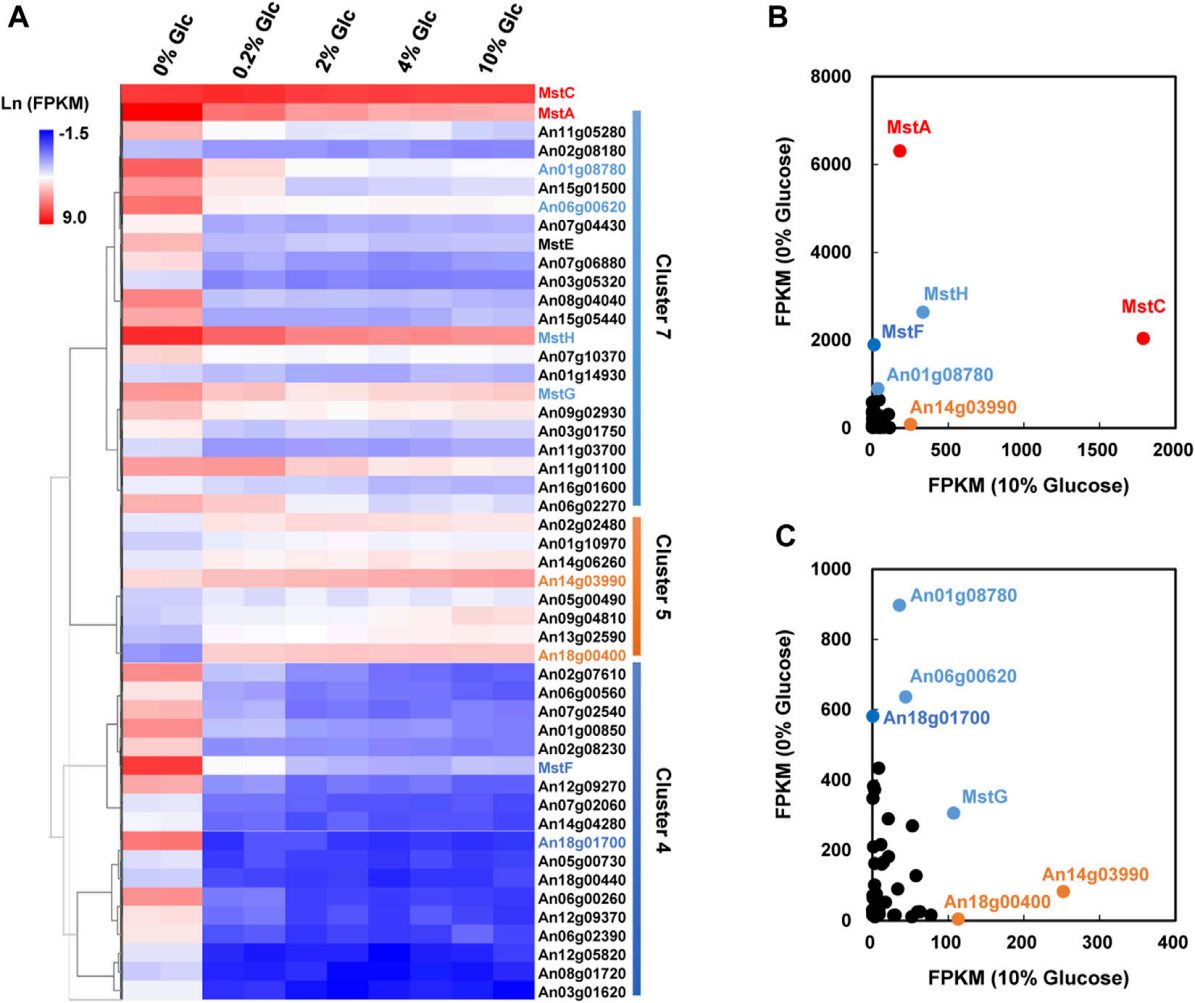
Transcriptional response of sugar transporters to glucose gradient in *Aspergillus niger*. **(A)** Hierarchical clustering analysis of 48 differentially expressed sugar transporters with FPKM>10 under at least one tested condition and the low-affinity glucose transporter MstC. Natural transformed gene expression values (FPKM) were color-coded from blue to red. These three distinguished clusters of sugar transporters were in line with their co-expression patterns identified by Fuzz cluste ring, including Cluster 4, Cluster 5, and Cluster 7, respectively. **(B,C)** Scatter plots of sugar transporters under no glucose and plentiful glucose (10%) conditions. The high-affinity glucose transporter MstA and low-affinity glucose transporter MstC showed the dominant expressions under the condition of no-carbon and 10% glucose, respectively. MstA and MstC were represented in red. The other sugar transporters with higher FPKM values were also indicated by their gene symbols in the same color as their corresponding clusters.

As mentioned above, carbohydrate uptake and assimilation were one of the most sensitive responses to external glucose fluctuation. According to the Pfam family of “Sugar and other transporters” (PF00083), 106 sugar transporters have been predicted in the genome of *A. niger.* Among these transporters, 48 were identified as DEGs under the various glucose concentrations in *A. niger* D353. Clustering analysis grouped 22 sugar transporters into Cluster 7, comprising four high-affinity glucose transporters, *mstA*, *mstE*, *mstG,* and *mstH* (Figure 4). These genes showed strong repression to external glucose increase and were greatly activated by glucose depletion. Comparably, other 18 sugar transporters in Cluster 4 were more significantly influenced by glucose changes, including another high-affinity glucose transporter *mstF*, which was dramatically inhibited by the addition of glucose and induced up to 166.99-fold under the condition of glucose depletion (Figure 4).

In consideration of gene expression FPKM value, *mstA*, *mstH*, *mstF, mstG,* An01g08780, An06g00620, and An18g01700 showed high expression when glucose was depleted (Figure 4), indicating these genes played curial roles for sugar uptake under the condition of no-carbon availability. In contrast, we also found 8 sugar transporters displayed different expression patterns, which belonged to Cluster 5 and were upregulated along with the increase of glucose addition. Especially, An14g03990 and An18g00400 displayed high expression at the glucose level of 10%, suggesting these two transporters may have the potential application at the plentiful carbon fermentation conditions. As to the low-affinity glucose transporter, different from the previous cognition of other filamentous fungi, the transcription of the *mstC* gene was glucose independent in *A. niger*, which maintained a high expression level at all tested conditions (Figure 4).

To further verify the response of glucose transporters to glucose, we constructed *in situ* labeling mutants of the low-affinity glucose transporter MstC and the high-affinity glucose transporters MstA in *A. niger* D353.8. The red fluorescent protein mCherry was fused to the C-terminal of these two selected transporters with a flexible linker ((G_4_S)_3_) by an approach of precise genome editing mediated by the CRISPR/Cas9 system (Zheng et al., 2019). For each transporter, 8 primary transformants with observed fluorescence were randomly picked up, purified, and genotypically verified by diagnostic PCR and DNA sequencing of fused open reading frames. It demonstrated that all these isolates were confirmed with the expected genotype (Supplementary Figure S2). Among them, the isolates KYD1.4, and KYD2.3 were selected for fluorescence imaging analysis of the *mCherry* labelling transporters MstA-mCherry, and MstC-mCherry, respectively.

**FIGURE 5:**
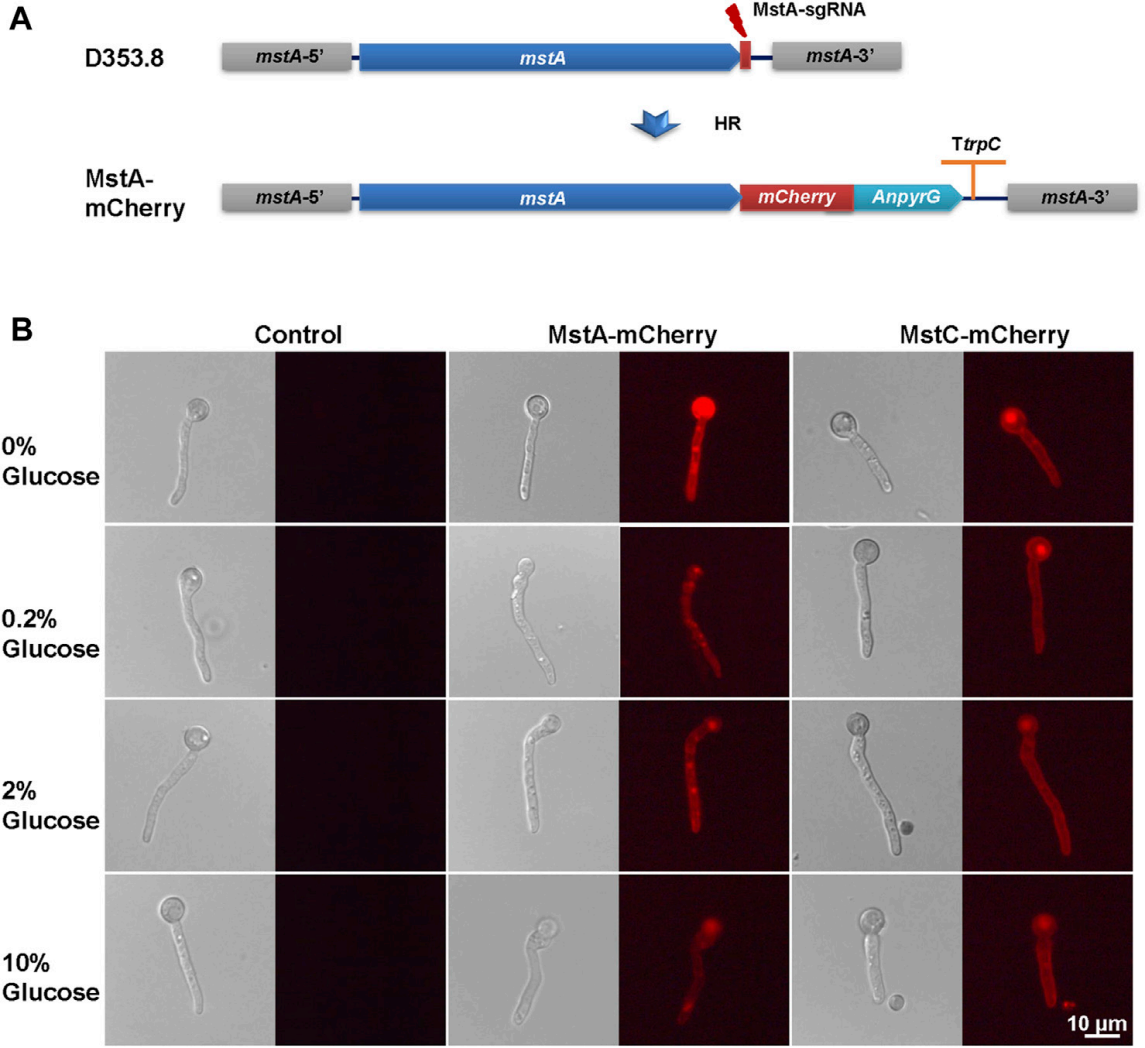
*In situ* fluorescence image analysis of MstA and MstC in *Aspergillus niger* with external glucose changes. **(A)** Schematic diagram of *mCherry* labeling glucose transporters construction. The donor DNAs were co-transformed with linear sgRNA mstA-sgRNA and Cas9 expression cassette into the protoplasts of *Aspergillus niger* D353.8. DNA double-strand breaks (DSBs) at 3′-flanking sequences of the *mstA* gene were generated by the Cas9 under the guide of sgRNAs and then were repaired by homologous recombination with the integration of donor DNAs containing *mCherry* and selection marker. **(B)** *In situ* fluorescence image analysis of *mCherry* labeling glucose transporters using a fluorescence microscope with the same settings. Representative images are given for each strain/glucose concentration. The scale bar represents 10 μm.

To observe the influence of glucose supply on subcellular localization and *in situ* fluorescence intensity of glucose transporters, the germinated conidia of isolates were induced in MM with different glucose concentrations for 1 h and then used for fluorescence imaging. As shown in Figure 5, MstA and MstC were observed to localize at the cellular cortex as integral proteins of the plasma membrane. Consistent with transcription profiling, their fluorescence intensity displayed similar trends in responding to external glucose changes (Figure 5). The high-affinity glucose transporters MstA showed the strongest fluorescence intensity in the absence of glucose, while its fluorescence faded away along with the increase of glucose concentration. By contrast, the MstC-mCherry maintained constant fluorescence intensity under all tested glucose conditions, which confirmed that the MstC expression was independent of glucose. These results suggested that these two classes of glucose transporters played distinct physiological functions: the low-affinity glucose transporter MstC ensured the basal glucose uptake with steady expression, while the high-affinity glucose transporter MstA might be kept in reserve for emergency use to cope with carbon starvation.

### 3.5 Effect of glucose gradients on citric acid metabolism and transport

**FIGURE 6:**
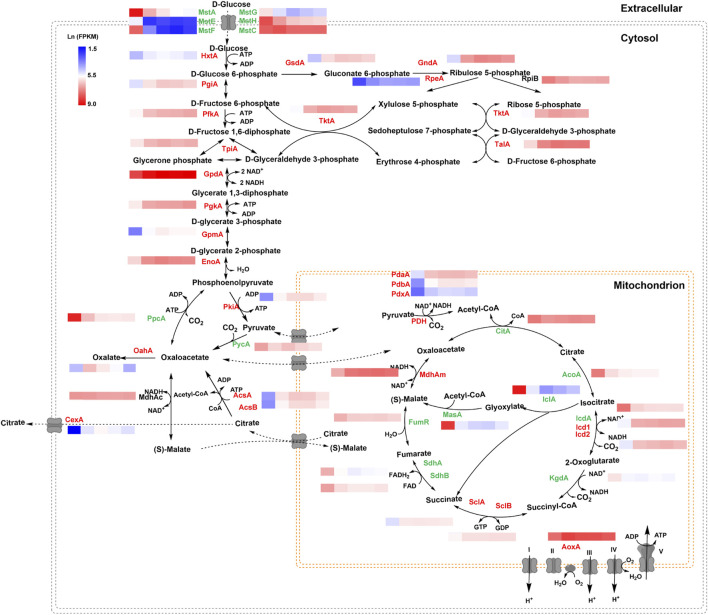
Transcriptional profiling of genes involved in the central metabolic pathway of *Aspergillus niger* under different glucose conditions. Natural transformed gene expression values (FPKM) were color-coded from blue to red. The genes labeled in green were downregulated along with the increase in glucose level, while the genes labeled in red were upregulated when glucose was increased. The genes labeled in black showed no significant change.

To elucidate the transcription changes under varying glucose conditions, the expression of genes involved in citric acid metabolism and transport was analyzed in detail (Figure 6). Along with the increase of glucose concentration, the expression of most of the genes in glycolysis and pentose phosphate pathway were upregulated, especially two genes using glucose-6-phosphate as substrate, glucose 6-phosphate isomerase gene *pgiA* and glucose 6-phosphate dehydrogenase gene *gsdA*. By contrast, several genes involved in gluconeogenesis, including phosphoenolpyruvate carboxykinase gene *ppcA*, and two genes in glyoxylate bypass, iso-citrate lyase *iclA* and malate synthase *masA* were greatly induced when glucose was depleted, as the anaplerotic reactions to cope with carbon starvation. In addition, most genes involved in TCA cycle were upregulated by glucose depletion, especially the cis-aconitase gene *acoA* and isocitrate dehydrogenase *icdA*. These data are consistent with glucose acting as a major signal to switch intracellular metabolisms to adapt the external carbon availability.

**FIGURE 7:**
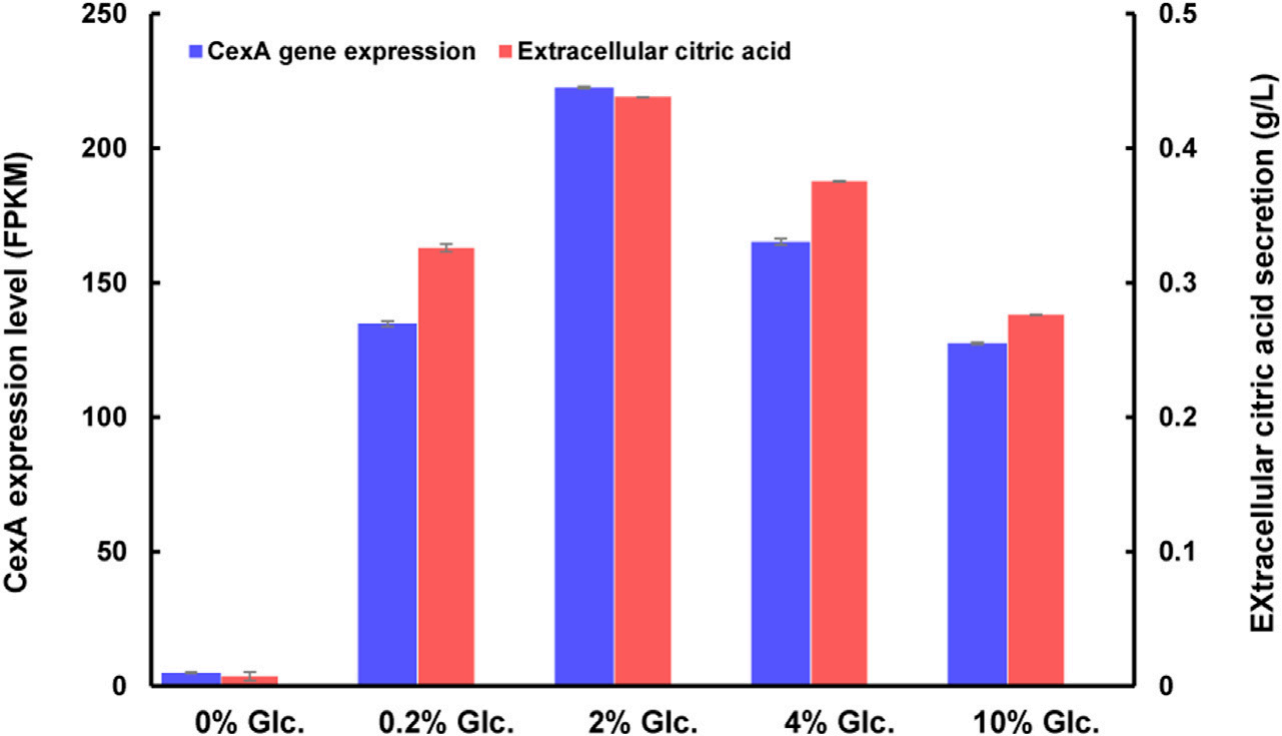
CexA transcriptional changes and extracellular citric acid concentrations in response to varying glucose concentrations. The gene expression intensity was represented as FPKM values. The extracellular citric acid titer of D353 mycelia, after being treated under different glucose concentrations, was detected by HPLC using a UV detector (Shimadzu, Kyoto, Japan) and Bio-Rad Aminex HPX-87H column (300 × 7.8 mm) (*n* = 3). The *cexA* gene expression and extracellular citric acid titer were shown in blue and red, respectively.

As to citric acid-related metabolism and transport, one major citric acid exporter CexA was considerately repressed with glucose withdrawal and gradually upregulated with the increase of glucose (Figure 7). To our knowledge, this is the first report where the transcriptional regulation of CexA in response to external glucose has been confirmed. To further analyze this finding, we measured extracellular citric acid concentrations by the approach of HPLC under varying glucose concentrations. The transcription of *cexA* was well-correlated with external citric acid concentrations (Figure 7), which were both virtually absent at 0% glucose, highest at 2%, and dropped slightly at the highest glucose levels used in this study (10%). These data are consistent with CexA being the major transporter of citric acid and indicate that citric acid titers during *A. niger* fermentation may be controlled at the level of *cexA* transcription.

## 4 Discussion

*Aspergillus niger* is an important industrial microorganism for the bulk bio-manufacturing of organic acids and enzymes, with good fitness to utilize a wide range of crude raw materials as carbon sources. To acclimate the nutrient fluctuation in both natural habitats and industrial processes, *A. niger* has developed complicated and fine-tuned sensing and signaling pathways for the coordination of extracellular nutrient availability with internal metabolism, growth, and production. As the preferred primary carbon source, glucose acts as a crucial signal to reshape intracellular physiological processes. Understanding the glucose signaling and responses, hence, has of great practical importance in industrial fermentation. Currently, however, no study has examined transcriptional responses of *A. niger* across multiple glucose concentrations, which constitutes a major knowledge gap in systems-level understanding of metabolic and cellular responses to this vital carbon source. In this study, we investigated the transcriptional regulation of *A. niger* mycelia in response to external glucose gradient using RNA-seq data. To break the limitation of comparative transcriptomic analysis between two samples, we conducted the Fuzzy c-mean clustering to unveil the global transcriptional pattern responses along with the increase of glucose levels. The glucose depletion led to reducing CCR and dramatically reprogrammed the expression of numerous genes and processes, including ribosomal biogenesis, carbon, and energy metabolisms.

The sugar signaling pathways play a crucial role in sensing, transporting, and transducing the external glucose stimulus (Zheng et al., 2022b). Generally, the cAMP/PKA signaling system is the major glucose sensing and signaling pathway in fungi (Zheng et al., 2022b). In *A. nidulans*, the putative GprD carbon receptor induced hyphal growth and conidial germination in the presence of glucose (Han et al., 2004), while the other GprH carbon and amino acid receptor activated glucose uptake during carbon starvation (Brown et al., 2015; Dos Reis et al., 2019). However, the orthologs of GprD or GprH in *A. niger* showed no detectable changes under different glucose conditions, suggesting the major components upstream of cAMP/PKA in *A. niger* were different from the one of *A. nidulans*. In contrast, GprM and GprF were significantly upregulated under carbon starvation, while the other two GPRCs, GprO and GprJ, showed reverse expression trends with induction by glucose (Table 1). As to the G-protein α-subunits, Lafon et al. reported that GanB was the key component of the cAMP/PKA pathway in response to glucose in *A. nidulans*, while the functional redundancy of the other two G-protein α-subunits FadA and GanA was not excluded (Han et al., 2004). In *A*. *niger*, similar to the GPRCs, the G-protein α-subunits also displayed different transcription patterns. As shown in Table 1, FadA has no significant transcriptional changes, but GanA was activated by the increase of glucose and GanB showed the opposite expression pattern, induced when glucose was depleted (Table 1). In addition, the G protein beta subunit SfaD and Gβ-like protein CpcB were also induced by glucose, which may interact with GanB to attenuate this signaling pathway. Similarly, the regulator of GanB signaling, RgsA, was activated by the increase of external glucose, which may strengthen the attenuation of this signaling in the presence of glucose, implying the GanB pathway was activated by carbon starvation and repressed by glucose supply. To sum up, these data suggested that glucose sensing and signaling were very complicated in *A. niger*, which might comprise two different glucose sensing and signaling pathways to respond to carbon starvation and glucose utilization.

The preference for glucose is also regulated by CCR in filamentous fungi (de Assis et al., 2021; Peng et al., 2021). In *Aspergilli*, four factors, CreA, CreB, CreC, and CreD, are involved in the regulation of CCR (Tanaka et al., 2017; Tanaka et al., 2018; Tanaka and Gomi, 2021). Among these factors, CreA was the major transcription factor to repress the expression of genes involved in alternative carbon utilization and sugar transport in a strong time and substrate composition dependent manner (Peng et al., 2018). In budding yeast, glucose depletion leads to the phosphorylation of Mig1, the ortholog of CreA, by active phosphorylated Snf1, resulting in the nuclear export-dependent degradation of Mig1 and the release of repression of alternative carbon sources hydrolases (Jamsheer et al., 2021; Caligaris et al., 2023; Correa-Romero et al., 2023). However, in *A. nidulans*, SnfA has not been identified to be associated with the phosphorylation of CreA (de Assis et al., 2018; de Assis et al., 2021). In this study, we also observed that the transcription of CreA, and SnfA remained stable in a glucose independent manner. Otherwise, a glycogen synthase kinase GskA, which has been reported to interact with CreA in *A. nidulans* (de Assis et al., 2021), was found to be dramatically upregulated by the presence of glucose in *A. niger*, which might be involved in the phosphorylation of CreA and the CCR regulation in response to glucose. CreB is a deubiquitinating enzyme and interacts with CreC (Espeso et al., 1995). CreD acts as an arrestin-related trafficking adaptor (ART) protein and interacts with HulA, involving in glucose-induced endocytosis (Tanaka et al., 2017). In *Aspergillus oryzae*, Tanaka et al. reported that the disruption of *creB* and *creC* caused a significant decrease in CreA when glucose was present (Tanaka et al., 2018). Whereas, in *A. nidulans*, it has been proven that the CreB deubiquitinating enzyme does not directly target CreA (Alam et al., 2017). Moreover, the CreA stability was not influenced by the *creD* disruption (Tanaka et al., 2017). and was not affected by the fluctuation of glucose, while the *creB* and *creD* showed opposite expression patterns, belonging to Cluster 4 and Cluster 2, respectively in *A. niger* (Table 2). These data implied that the glucose response of CreA-mediated CCR might not be dependent on the transcription of CreA or there might be other as yet unknown factors controlled by CreB and CreD involved in the CreA-independent CCR. Further investigation of CreA nuclear export-dependent degradation and identification of CreB/CreD targets would deepen the understanding of the CCR regulation mechanism in filamentous fungi.

In response to external glucose fluctuation, the genes involved in carbohydrate assimilation were dramatically regulated, including a large number of hydrolytic enzymes and sugar transporters. Especially, when glucose is depleted, 52 CAZy enzymes and their regulators of carbon sources, including starch, cellulose, and xylan, were considerably activated, due to the release of CCR (Table 2; Supplementary Table S5). This fitness to carbon starvation is in line with the rapid degradation and uptake of available carbon nutrition. In addition to carbon hydrolytic enzymes, 48 sugar transporters also transcriptionally responded. Consistent with the glucose regulation of high-affinity glucose transporter in other fungi *N. crassus* (Wang et al., 2017), several high-affinity glucose transporters, *mstA*, *mstE*, *mstF*, *mstG,* and *mstH*, were significantly upregulated by glucose absence in *A. niger*. However, the effect of glucose on the transcription of a low-affinity glucose transport system was distinguished into two species. The transcript of low-affinity glucose transporter Glt1 was decreased by carbon starvation, and induced in a glucose dosage-dependent pattern in *Neurospora crassus* (Wang et al., 2017). By contrast, the expression of low-affinity glucose transporter MstC was independent of glucose gradient, which remained stable mRNA levels under glucose variations in *A. niger*. Instead, the MstC regulation was proven at the protein level, by *in situ* fluorescence labeling assay (Figure 5). These data might indicate that low-affinity glucose transporter MstC functions in a glucose-independent way to ensure the fundamental glucose uptake in *A. niger*, while the high-affinity glucose transport system mainly focused on the response of carbon starvation.

In addition to carbon metabolism and uptake, we also observed that citric acid efflux was dramatically influenced by external glucose. The citric acid exporter CexA was almost totally repressed when glucose was depleted (Figure 6), resulting in no detectable extracellular citric acid (Figure 7). Compared with the condition of no glucose, the transcription of CexA was greatly induced up to 27.06-fold and 44.68-fold at the glucose level of 0.2% and 2%, respectively. Based on this glucose-dependent regulation of CexA, an adaptive evolutionary hypothesis of *A. niger* could be proposed: when the external glucose, even very low amount, is available in the biotope, *A. niger* rapidly accumulates citric acid and secretes this molecule into the surroundings, resulting in the acidification of the biotope to gain competitive advantage over neutrophilic organisms; but when glucose is depleted, the citric acid export is immediately shut down, to maintain the energy of cell survival. As to the transcription regulation of CexA, a putative methyltransferase LaeA has been reported to be involved in the regulation of CexA in the white koji fungus *Aspergillus luchuensis mut. Kawachii* (Kadooka et al., 2020). However, the LaeA of *A. niger* displayed the opposite expression pattern of CexA, which was only activated in the absence of glucose, although such chromatin remodelers may not be mainly regulated at the level of transcription. Therefore, further investigation of glucose-dependent transcription regulation of CexA is of both physiological and industrial importance.

## 5 Conclusion

In this study, the transcriptional regulation of *A. niger* in response to glucose gradient was comprehensively analyzed based on RNA-seq data. It demonstrated that external glucose fluctuation led to transcriptional reprogramming of considerable genes, including ribosomal biogenesis, carbon transport and catabolism, glucose sensing, and signaling in *A. niger*. As to CCR regulation, it indicated that CreA-mediated CCR was not dependent on the transcription of CreA, while CreB and CreD were significantly regulated by the glucose level, suggesting they might involve in the regulation of CreA-independent CCR. Transporters were one of the most sensitive elements to respond to the glucose variance. Several high-affinity glucose transporters were activated by glucose depletion for the maximum absorption of environmental glucose, while the low-affinity glucose transporter MstC remains high expression in a glucose-independent pattern to ensure the basal glucose uptake in *A. niger*. Fluorescent microscopy demonstrated clear concordance of MstA and MstC protein abundance with respective gene expression profiles, thus providing proof of the principle that transcriptional analysis in this study will be valuable for understanding other *A. niger* membrane transporters in future studies. In addition, it is interesting to observe that the citric acid efflux was also regulated by glucose gradient, which could shed light on the adaptive evolutionary mechanism of citric acid production of *A. niger*. Taken together, given the complexity of glucose sensing and signaling, the transcriptional responses of glucose gradient unveiled in this study, shed new light on the understanding of physiological regulation and evolutionary mechanism of this industrially important fungal cell factory.

## Data Availability

The original contributions presented in the study are included in the article/Supplementary Material, further inquiries can be directed to the corresponding authors.
