# Supplementary material for: Genome-wide transcription landscape of citric acid producing Aspergillus niger in response to glucose gradient
Source: Front Bioeng Biotechnol. 2023 Oct 24;11:1282314. doi: 10.3389/fbioe.2023.1282314 (PMC10628723; doi:10.3389/fbioe.2023.1282314)
Supplement: Supplementary file 1 [file DataSheet1.zip › Data Sheet 1/2-Frontiers_Supplementary_Material/Supplementary Figure S2.docx]

Genome-wide transcription landscape of citric acid producing *Aspergillus niger* in response to glucose gradient

Xiaomei Zheng^1,2,3,4†^, Peng Du^1,2^, Kaiyue Gao^1,2^, Yimou Du^1,2^, Timothy C. Cairns^5†^, Xiaomeng Ni^1,2,3^, Meiling Chen^2,6^, Wei Zhao^7^, Xinrong Ma^1*^, Hongjiang Yang^1*^, Ping Zheng^1,2,3,4†*^, and Jibin Sun^1,2,3,4†^

^1^College of Biotechnology, Tianjin University of Science & Technology, Tianjin, China

^2^Tianjin Institute of Industrial Biotechnology, Chinese Academy of Sciences, Tianjin, China

^3^National Technology Innovation Center of Synthetic Biology, Tianjin China

^4^University of Chinese Academy of Sciences, Beijing, China

^5^Chair of Applied and Molecular Microbiology, Institute of Biotechnology, Technische Universität Berlin, Berlin, Germany

^6^School of Biotechnology, East China University of Science and Technology, Shanghai 200237, China

^7^Shan Dong Fuyang Biological Technology Co., Ltd, Dezhou 253100, China

^†^ORCID:

Xiaomei Zheng: zheng_xm@tib.cas.cn, ORCID: 0000-0001-9136-0666;

Timothy C. Cairns: t.cairns@tu-berlin.de, ORCID: 0000-0001-7106-224X;

Ping Zheng: zheng_p@tib.cas.cn, ORCID: 0000-0001-9434-9892;

Jibin Sun: sun_jb@tib.cas.cn, ORCID: 0000-0002-0208-504X.

*** Correspondence:**Xinrong Ma
xinrong.ma@tust.edu.cn

Hongjiang Yang
hongjiangyang@tust.edu.cn

Ping Zheng
zheng_p@tib.cas.cn

**Supplementary Figure S2**


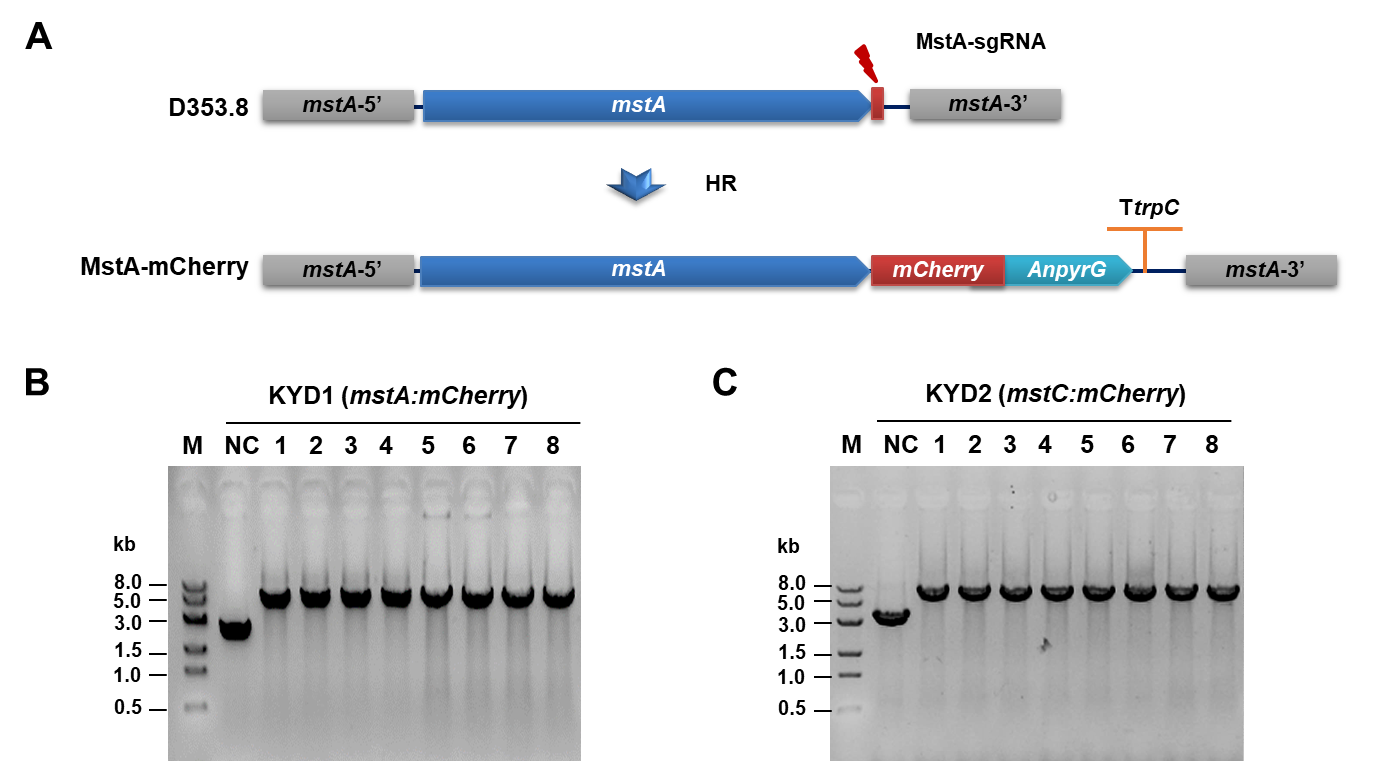


# Figure S2. Construction of *in situ* labeling mutants of *mstA* and *mstC* fused with fluorescent protein mCherry at the C-terminal in *A. niger*

A. Schematic diagram of *in situ* fluorescent protein mCherry labeling design of glucose transporters *mstA* and *mstC* in *A.niger* D353.8. The donor DNAs were co-transformed with linear sgRNA constructs (such as MstA-sgRNA2) and Cas9 expression cassette into the protoplasts of *A. niger* D353.8. Two DNA double-strand breaks (DSBs) at the flanking sequences of the glucose transporter encoding genes were generated by the Cas9 under the guide of sgRNAs, and then were repaired by HR with the integration of donor DNAs. B and C. Diagnostic PCR analysis of primary transformants of *in situ* fluorescent protein mCherry labeling mutants. When the donor DNAs harboring *mCherry*:*pyrG:TtrpC* DNA fragments were correctly integrated into the genomic loci of glucose transporter encoding sequences, the expected sizes of PCR products with the verified primers of *mstA:mCherry* and *mstC:mCherry* were 3724-bp, and 3401-bp, respectively. The parent strain D353.8 was used as negative control, which was represented as “NC” in each electrophoretogram. For each mutant, the verified positive transformants were selected for further subcellular localization and fluorescence intensity detection.
