# Supplementary material for: Genome-wide transcription landscape of citric acid producing Aspergillus niger in response to glucose gradient
Source: Front Bioeng Biotechnol. 2023 Oct 24;11:1282314. doi: 10.3389/fbioe.2023.1282314 (PMC10628723; doi:10.3389/fbioe.2023.1282314)
Supplement: Supplementary file 1 [file DataSheet1.zip › Data Sheet 1/2-Frontiers_Supplementary_Material/Supplementary Table S1.docx]

Genome-wide transcription landscape of citric acid producing *Aspergillus niger* in response to glucose gradient

Xiaomei Zheng^1,2,3,4†^, Peng Du^1,2^, Kaiyue Gao^1,2^, Yimou Du^1,2^, Timothy C. Cairns^5†^, Xiaomeng Ni^1,2,3^, Meiling Chen^2,6^, Wei Zhao^7^, Xinrong Ma^1*^, Hongjiang Yang^1*^, Ping Zheng^1,2,3,4†*^, and Jibin Sun^1,2,3,4†^

^1^College of Biotechnology, Tianjin University of Science & Technology, Tianjin, China

^2^Tianjin Institute of Industrial Biotechnology, Chinese Academy of Sciences, Tianjin, China

^3^National Technology Innovation Center of Synthetic Biology, Tianjin China

^4^University of Chinese Academy of Sciences, Beijing, China

^5^Chair of Applied and Molecular Microbiology, Institute of Biotechnology, Technische Universität Berlin, Berlin, Germany

^6^School of Biotechnology, East China University of Science and Technology, Shanghai 200237, China

^7^Shan Dong Fuyang Biological Technology Co., Ltd, Dezhou 253100, China

^†^ORCID:

Xiaomei Zheng: zheng_xm@tib.cas.cn, ORCID: 0000-0001-9136-0666;

Timothy C. Cairns: t.cairns@tu-berlin.de, ORCID: 0000-0001-7106-224X;

Ping Zheng: zheng_p@tib.cas.cn, ORCID: 0000-0001-9434-9892;

Jibin Sun: sun_jb@tib.cas.cn, ORCID: 0000-0002-0208-504X.

*** Correspondence:**Xinrong Ma
xinrong.ma@tust.edu.cn

Hongjiang Yang
hongjiangyang@tust.edu.cn

Ping Zheng
zheng_p@tib.cas.cn

**Supplementary Table S1**

**Table S1 Strains and plasmids used in the study.**

| **Strains/plasmids** | **Description** | **Reference** |
| --- | --- | --- |
| *A.niger* D353 | Citric acid producing strain | (Zhang, et al., 2020) |
| *A.niger* D353.8 | *pyrG::Hph, Hyg^R^* | (Zhang, et al., 2020) |
| KYD1.4 | D353.8, *mstA*:*mCherry*:*pyrG* | This study |
| KYD2.3 | D353.8, *mstC*:*mCherry*:*pyrG* | This study |
| pCas9-AnpyrG | P*glaA*:*Cas9*:T*glaA*, *Hyg^R^* | (Zhang, et al., 2020) |
| psgRNA6.18 | P*5S rRNA*:agdA-sgRNA1:T*poly(T)_6_* | (Lu, et al., 2022) |
| psgRNA6.21 | P*5S rRNA*:mstA-sgRNA2:T*poly(T)_6_* | This study |
| psgRNA6.23 | P*5S rRNA*:mstC-sgRNA2:T*poly(T)_6_* | This study |

**Reference**

Lu, Y., Zheng, X., Wang, Y., Zhang, L., Wang, L., Lei, Y., et al. (2022) Evaluation of Aspergillus niger Six Constitutive Strong Promoters by Fluorescent-Auxotrophic Selection Coupled with Flow Cytometry: A Case for Citric Acid Production, *Journal of fungi (Basel, Switzerland)* **8**.

Zhang, L., Zheng, X., Cairns, T.C., Zhang, Z., Wang, D., Zheng, P., and Sun, J. (2020) Disruption or reduced expression of the orotidine-5'-decarboxylase gene pyrG increases citric acid production: a new discovery during recyclable genome editing in Aspergillus niger, *Microbial cell factories* **19**: 76.
