# Supplementary material for: Genome-wide transcription landscape of citric acid producing Aspergillus niger in response to glucose gradient
Source: Front Bioeng Biotechnol. 2023 Oct 24;11:1282314. doi: 10.3389/fbioe.2023.1282314 (PMC10628723; doi:10.3389/fbioe.2023.1282314)
Supplement: Supplementary file 1 [file DataSheet1.zip › Data Sheet 1/2-Frontiers_Supplementary_Material/Supplementary Table S3.docx]

Genome-wide transcription landscape of citric acid producing *Aspergillus niger* in response to glucose gradient

Xiaomei Zheng^1,2,3,4†^, Peng Du^1,2^, Kaiyue Gao^1,2^, Yimou Du^1,2^, Timothy C. Cairns^5†^, Xiaomeng Ni^1,2,3^, Meiling Chen^2,6^, Wei Zhao^7^, Xinrong Ma^1*^, Hongjiang Yang^1*^, Ping Zheng^1,2,3,4†*^, and Jibin Sun^1,2,3,4†^

^1^College of Biotechnology, Tianjin University of Science & Technology, Tianjin, China

^2^Tianjin Institute of Industrial Biotechnology, Chinese Academy of Sciences, Tianjin, China

^3^National Technology Innovation Center of Synthetic Biology, Tianjin China

^4^University of Chinese Academy of Sciences, Beijing, China

^5^Chair of Applied and Molecular Microbiology, Institute of Biotechnology, Technische Universität Berlin, Berlin, Germany

^6^School of Biotechnology, East China University of Science and Technology, Shanghai 200237, China

^7^Shan Dong Fuyang Biological Technology Co., Ltd, Dezhou 253100, China

^†^ORCID:

Xiaomei Zheng: zheng_xm@tib.cas.cn, ORCID: 0000-0001-9136-0666;

Timothy C. Cairns: t.cairns@tu-berlin.de, ORCID: 0000-0001-7106-224X;

Ping Zheng: zheng_p@tib.cas.cn, ORCID: 0000-0001-9434-9892;

Jibin Sun: sun_jb@tib.cas.cn, ORCID: 0000-0002-0208-504X.

*** Correspondence:**Xinrong Ma
xinrong.ma@tust.edu.cn

Hongjiang Yang
hongjiangyang@tust.edu.cn

Ping Zheng
zheng_p@tib.cas.cn

**Supplementary Table S3**

**Table S3 Primers used in this study.**

| **Primer name** | **Primer Sequence (5' to 3')** |
| --- | --- |
| \| Primers to construct targeting sgRNA plasmids \| \| --- \| | |
| mstA-sgRNA2-F | caccCGGGATATCAGTTTCGACAA |
| mstA-sgRNA2-R | aaacTTGTCGAAACTGATATCCCG |
| mstC-sgRNA2-F | caccCCACGCACGCAATGTTTGAA |
| mstC-sgRNA2-R | aaacTTCAAACATTGCGTGCGTGG |
| \| Primers to construct donor DNAs for glucose transporters mCherry-labeling \| \| --- \| | |
| Mhi-mstA-mCherry-F | cgcttccctacgttgcgactttcccttctccttccctttcCAGGAAACAGCTATGAC |
| Mhi-mstA-mCherry-R | cggcgacgctcgcaatcacgtagatgttgccaatcaacatGGTGTTTAAACGGTGATGTC |
| Mhi-mstC-mCherry-F | tcggtttctctttcactgtgcagaccgtcacacagtttctCAGGAAACAGCTATGAC |
| Mhi-mstC-mCherry-R | gggcctcgacgcgcgaggcgtcaacgaagccttcagccatGGTGTTTAAACGGTGATGTC |
| Primers for diagnostic PCR of transformants | |
| mstA-g-F | GCGAGCCATCCCAATCAAGCC |
| mstA-g-R | GAAGACAAAGACACGGAACAGGT |
| mstC-g-F | TCACTCGTTGACACCGTCATC |
| mstC-g-R | CCCAACCATATCCTTCCCGATGA |

Notes: Restriction sites are underlined. Fm represents forward primer with modification and Rm represents reverse primer with modification. The modified additional sequences were represented in lowercase letters.
