# Supplementary material for: Genome-wide transcription landscape of citric acid producing Aspergillus niger in response to glucose gradient
Source: Front Bioeng Biotechnol. 2023 Oct 24;11:1282314. doi: 10.3389/fbioe.2023.1282314 (PMC10628723; doi:10.3389/fbioe.2023.1282314)
Supplement: Supplementary file 1 [file DataSheet1.zip › Data Sheet 1/2-Frontiers_Supplementary_Material/Supplementary Table S4.docx]

Genome-wide transcription landscape of citric acid producing *Aspergillus niger* in response to glucose gradient

Xiaomei Zheng^1,2,3,4†^, Peng Du^1,2^, Kaiyue Gao^1,2^, Yimou Du^1,2^, Timothy C. Cairns^5†^, Xiaomeng Ni^1,2,3^, Meiling Chen^2,6^, Wei Zhao^7^, Xinrong Ma^1*^, Hongjiang Yang^1*^, Ping Zheng^1,2,3,4†*^, and Jibin Sun^1,2,3,4†^

^1^College of Biotechnology, Tianjin University of Science & Technology, Tianjin, China

^2^Tianjin Institute of Industrial Biotechnology, Chinese Academy of Sciences, Tianjin, China

^3^National Technology Innovation Center of Synthetic Biology, Tianjin China

^4^University of Chinese Academy of Sciences, Beijing, China

^5^Chair of Applied and Molecular Microbiology, Institute of Biotechnology, Technische Universität Berlin, Berlin, Germany

^6^School of Biotechnology, East China University of Science and Technology, Shanghai 200237, China

^7^Shan Dong Fuyang Biological Technology Co., Ltd, Dezhou 253100, China

^†^ORCID:

Xiaomei Zheng: zheng_xm@tib.cas.cn, ORCID: 0000-0001-9136-0666;

Timothy C. Cairns: t.cairns@tu-berlin.de, ORCID: 0000-0001-7106-224X;

Ping Zheng: zheng_p@tib.cas.cn, ORCID: 0000-0001-9434-9892;

Jibin Sun: sun_jb@tib.cas.cn, ORCID: 0000-0002-0208-504X.

*** Correspondence:**Xinrong Ma
xinrong.ma@tust.edu.cn

Hongjiang Yang
hongjiangyang@tust.edu.cn

Ping Zheng
zheng_p@tib.cas.cn

**Supplementary Table S4**

**Table S4 Summary of RNA sequencing and mapping in this study.**

| **Sample** | **GC Content** | **%≥Q30** | **Total Reads** | **Mapped**  **Reads** | **Uniq Mapped Reads** | **Multiple Map Reads** |
| --- | --- | --- | --- | --- | --- | --- |
| 0%Glc-1 | 54.18% | 94.33% | 43,823,072 | 39,067,134 (89.15%) | 37,968,643 (86.64%) | 1,098,491 (2.51%) |
| 0%Glc-2 | 54.19% | 94.71% | 41,935,780 | 37,842,803 (90.24%) | 36,816,058 (87.79%) | 1,026,745 (2.45%) |
| 0.2%Glc-1 | 54.75% | 94.67% | 43,090,518 | 39,373,980 (91.38%) | 38,456,156 (89.25%) | 917,824 (2.13%) |
| 0.2%Glc-2 | 54.61% | 92.79% | 53,247,914 | 48,461,078 (91.01%) | 47,084,703 (88.43%) | 1,376,375 (2.58%) |
| 2%Glc-1 | 54.87% | 94.60% | 42,672,258 | 39,159,770 (91.77%) | 38,345,901 (89.86%) | 813,869 (1.91%) |
| 2%Glc-2 | 54.69% | 92.63% | 60,131,104 | 54,869,548 (91.25%) | 52,924,293 (88.01%) | 1,945,255 (3.24%) |
| 4%Glc-1 | 54.84% | 94.11% | 39,902,834 | 36,415,412 (91.26%) | 35,706,742 (89.48%) | 708,670 (1.78%) |
| 4%Glc-2 | 54.69% | 92.38% | 56,617,506 | 51,424,831 (90.83%) | 50,237,914 (88.73%) | 1,186,917 (2.10%) |
| 10%Glc-1 | 54.72% | 95.41% | 42,275,696 | 38,861,030 (91.92%) | 38,126,627 (90.19%) | 734,403 (1.74%) |
| 10%Glc-2 | 54.90% | 95.25% | 46,024,360 | 42,248,745 (91.80%) | 41,322,583 (89.78%) | 926,162 (2.01%) |
