# Supplementary material for: Genome-wide transcription landscape of citric acid producing Aspergillus niger in response to glucose gradient
Source: Front Bioeng Biotechnol. 2023 Oct 24;11:1282314. doi: 10.3389/fbioe.2023.1282314 (PMC10628723; doi:10.3389/fbioe.2023.1282314)
Supplement: Supplementary file 1 [file DataSheet1.zip › Data Sheet 1/2-Frontiers_Supplementary_Material/Supplementary Table S5.docx]

Genome-wide transcription landscape of citric acid producing *Aspergillus niger* in response to glucose gradient

Xiaomei Zheng^1,2,3,4†^, Peng Du^1,2^, Kaiyue Gao^1,2^, Yimou Du^1,2^, Timothy C. Cairns^5†^, Xiaomeng Ni^1,2,3^, Meiling Chen^2,6^, Wei Zhao^7^, Xinrong Ma^1*^, Hongjiang Yang^1*^, Ping Zheng^1,2,3,4†*^, and Jibin Sun^1,2,3,4†^

^1^College of Biotechnology, Tianjin University of Science & Technology, Tianjin, China

^2^Tianjin Institute of Industrial Biotechnology, Chinese Academy of Sciences, Tianjin, China

^3^National Technology Innovation Center of Synthetic Biology, Tianjin China

^4^University of Chinese Academy of Sciences, Beijing, China

^5^Chair of Applied and Molecular Microbiology, Institute of Biotechnology, Technische Universität Berlin, Berlin, Germany

^6^School of Biotechnology, East China University of Science and Technology, Shanghai 200237, China

^7^Shan Dong Fuyang Biological Technology Co., Ltd, Dezhou 253100, China

^†^ORCID:

Xiaomei Zheng: zheng_xm@tib.cas.cn, ORCID: 0000-0001-9136-0666;

Timothy C. Cairns: t.cairns@tu-berlin.de, ORCID: 0000-0001-7106-224X;

Ping Zheng: zheng_p@tib.cas.cn, ORCID: 0000-0001-9434-9892;

Jibin Sun: sun_jb@tib.cas.cn, ORCID: 0000-0002-0208-504X.

*** Correspondence:**Xinrong Ma
xinrong.ma@tust.edu.cn

Hongjiang Yang
hongjiangyang@tust.edu.cn

Ping Zheng
zheng_p@tib.cas.cn

**Supplementary Table S5**

**Table S5** **Differentially expressed CAZy genes in response to external glucose**

| **Substrate** | **Gene ID** | **Name** | **CAZy family** | **Regulator** | **Mfuzz**  **Cluster** | **0% Glc.** | **0.2% Glc.** | **2% Glc.** | **4% Glc.** | **10% Glc.** |
| --- | --- | --- | --- | --- | --- | --- | --- | --- | --- | --- |
| starch | An02g13240 | *agdC* | GH13 |  | 4 | 73.79±0.7 | 9.29±2.92 | 8.08±0.01 | 9.42±0.58 | 12.96±1.62 |
| starch | An11g03340 | *aamA* | GH13 | AmyR | 4 | 1590.79±34.51 | 449.01±109.64 | 323.94±66.15 | 432.98±55.73 | 556.59±10.11 |
| starch | An05g02100 | *amyA* | GH13 |  | 4 | 5120.04±94.93 | 1341.54±140.11 | 996.85±76.23 | 1015.08±35.04 | 1192.6±10.64 |
| starch | An12g06930 | *amyA* | GH13 |  | 4 | 4936.17±75.79 | 1304.28±134.92 | 965.76±74.63 | 985.05±37.35 | 1152.67±7.26 |
| starch | An04g06930 | *amyC* | GH13 | AmyR | 4 | 165.78±1.92 | 22.2±4.46 | 17.83±0.59 | 16.57±0.01 | 17.29±0.25 |
| starch | An01g06120 | *gdbA* | GH133 |  | 4 | 58.64±0.1 | 23.13±6.27 | 18.15±1.62 | 20.98±0.25 | 27.23±1.6 |
| starch | An03g06550 | *glaA* | GH15 | AmyR, AraR | 7 | 10016.16±  148.66 | 7024.22±  95.34 | 5175.41±  290.25 | 4557.5±  105.32 | 5450.05±  15.63 |
| starch | An12g03070 | *glaB* | GH15 |  | 6 | 65.65±1.91 | 16.07±1.19 | 17.46±1.26 | 22.84±0.46 | 44.15±1.17 |
| starch | An04g06920 | *agdA* | GH31 | AmyR | 4 | 1982.84±9.65 | 961.16±82.18 | 729.6±10.62 | 766.14±6.84 | 983.42±20.3 |
| starch | An01g10930 | *agdB* | GH31 | AmyR | 4 | 759.94±23.66 | 108.88±40.8 | 77.18±3.24 | 75.23±1.04 | 94.21±0.75 |
| starch | An09g05880 | *agdE* | GH31 |  | 4 | 108.13±0.34 | 35.8±2.51 | 34.04±0.88 | 35.19±0.09 | 29.85±0.37 |
| xylan | An01g09960 | *xlnD* | GH3 | XlnR | 4 | 12.89±0.26 | 1.44±0.31 | 0.92±0.01 | 0.79±0.02 | 0.9±0.22 |
| xylan | An08g10780 | *gbgA* | GH43 | AraR | 4 | 144.16±2.54 | 50.6±2.6 | 40.62±0.35 | 39.68±0.64 | 59.27±1.95 |
| xylan | An11g03120 | *xynD* | GH43 |  | 4 | 46.13±0.42 | 7.2±1.73 | 3.72±0.43 | 4.66±0.62 | 5.45±0.52 |
| xyloglucan | An01g03340 | *xgeA* | GH12 |  | 1 | 15.9±0.98 | 11.11±0.2 | 10.4±0.57 | 12.3±0.36 | 11.81±0.48 |
| xyloglucan | An14g02760 | *xgeB* | GH12 |  | 4 | 12.05±0.31 | 30.16±0.21 | 18.26±0.8 | 18.27±1.83 | 17.18±0.4 |
| xyloglucan | An09g03300 | *axlA* | GH31 | AraR, XlnR | 5 | 4.57±0.23 | 8.01±0.79 | 8.32±0.15 | 9.82±0.15 | 12.12±0.85 |
| xyloglucan | An01g04880 | *axlB* | GH31 |  | 4 | 46.92±0.59 | 7.45±2.72 | 6.74±0.16 | 5.79±0.17 | 5.2±0.37 |
| xyloglucan | An16g02760 | *afcA* | GH95 | XlnR | 4 | 11.69±0.02 | 3.67±1.9 | 2.65±0.42 | 2.78±0.21 | 3.09±0.08 |
| cellulose | An03g03740 | *bgl4* | GH1 |  | 4 | 88.37±0.09 | 32.91±2.3 | 28.42±1.9 | 28.19±0.01 | 30.7±1.33 |
| cellulose | An08g04630 |  | GH131 |  | 4 | 11.01±1.38 | 9.88±0.95 | 7.75±0.85 | 9.18±0.61 | 7.68±0.51 |
| cellulose | An18g03570 | *bglA* | GH3 | XlnR | 4 | 899.12±1.92 | 11.9±0.37 | 5.6±0.17 | 5.43±0.3 | 5.23±0.3 |
| cellulose | An03g05330 |  | GH3 |  | 4 | 16.83±0.93 | 4.63±0.49 | 3.71±0 | 4.33±0.21 | 4.71±0.03 |
| cellulose | An11g06080 |  | GH3 |  | 4 | 34.12±0.11 | 7.63±0.04 | 6±1.07 | 4.54±0.27 | 5.25±0.65 |
| cellulose | An14g01770 |  | GH3 | AmyR | 4 | 39.85±0.06 | 3±1.22 | 2.33±0.12 | 2.68±0.16 | 3.03±0.01 |
| cellulose | An17g00520 |  | GH3 |  | 4 | 12.17±1.18 | 3.92±0.79 | 3.65±0.43 | 4.1±0.03 | 4.03±0.21 |
| cellulose | An16g06800 | *eglB* | GH5 | AraR | 5 | 119.1±3.22 | 156.95±2.58 | 149.29±12.84 | 223.25±12.6 | 188.82±1.22 |
| cellulose | An07g09330 | *cbhA* | GH7 |  | 4 | 11.52±0.01 | 1.33±0.37 | 0.98±0.33 | 1.24±0.23 | 0.73±0.05 |
| galacto-mannan | An12g01850 | *mndB* | GH2 | XlnR | 4 | 99.34±1.08 | 12.48±3.49 | 8.09±0.44 | 8±0.42 | 6.7±0.31 |
| galacto-mannan | An06g00170 | *aglA* | GH27 | AraR, AmyR | 4 | 41.87±0.27 | 1.94±2.32 | 1.1±0.09 | 1.25±0.26 | 0.94±0.07 |
| galacto-mannan | An09g00260 | *aglC* | GH36 |  | 7 | 39.88±0.22 | 28.5±2.48 | 20.05±0.93 | 8.34±0.14 | 8.22±0.66 |
| galacto-mannan | An09g00270 | *aglC* | GH36 | RhaR | 7 | 23.39±0.54 | 17.48±10.5 | 13.71±2.72 | 4.7±0.53 | 5.16±1.57 |
| galacto-mannan | An04g02700 |  | GH36 | AmyR | 4 | 32.57±0.36 | 12.76±3.67 | 11.11±0.81 | 11.98±0.92 | 14.18±0.32 |
| inulin | An12g08280 | *inuE* | GH32 | InuR | 4 | 54.21±1.02 | 0.36±0.1 | 0.24±0.18 | 0.27±0.12 | 0.15±0.09 |
| pectin | An09g02160 | rgaeA | CE12 | GaaR, RhaR | 4 | 11.24±0.04 | 1.74±0.28 | 2.5±0.37 | 2.12±0.01 | 2.48±0.37 |
| pectin | An04g09360 | rgaeB | CE12 | GaaR, RhaR | 4 | 11.46±0.23 | 2.03±0.4 | 1.47±0.16 | 1.65±0.03 | 1.32±0.23 |
| pectin | An04g09690 | pmeB | CE8 | GaaR, AraR | 5 | 4.81±0.6 | 8.23±0.24 | 8.79±0.2 | 10.93±0.06 | 12.18±0.72 |
| pectin | An14g02920 | urhgA | GH105 | AraR, GaaR, RhaR | 4 | 15.38±1.11 | 1.24±1.77 | 0.5±0.02 | 0.36±0.14 | 0.5±0.07 |
| pectin | An02g04900 | pgaB | GH28 | GaaR | 3 | 26.18±0.43 | 62.73±4.45 | 60.7±3.14 | 69.12±1.27 | 77.38±4.38 |
| pectin | An12g07500 | pgaX | GH28 | GaaR | 4 | 27.47±0.27 | 6.42±0.78 | 6.3±0.38 | 7.31±0.77 | 6.72±0.09 |
| pectin | An12g00950 | rhgA | GH28 |  | 5 | 5.61±0.28 | 7.46±0.07 | 10.25±0.15 | 14.48±0.38 | 13.81±0.06 |
| pectin | An09g01190 | abnA | GH43 | AraR, GaaR | 4 | 65.25±1.08 | 1.03±1.31 | 0.74±0.1 | 0.49±0.1 | 0.56±0.08 |
| pectin | An02g10550 | abnC | GH43 | XlnR | 4 | 34.57±0.07 | 3.42±1.21 | 2.8±0.11 | 1.44±0.27 | 1.1±0.2 |
| pectin | An16g02730 | abnD | GH43 | AmyR, GaaR, RhaR | 8 | 21.73±0.24 | 58.64±1.19 | 63.11±5.82 | 70.11±1.97 | 53.38±1.52 |
| pectin | An08g01710 | abfC | GH51 | AraR, GaaR | 4 | 133.18±1.23 | 3.59±3.67 | 1.49±0.19 | 0.9±0.18 | 1.22±0.01 |
| pectin | An15g02300 | abfB | GH54 | AraR | 4 | 256.8±2.16 | 0.33±53.03 | 0.14±0.1 | 0.17±0.01 | 0.26±0.13 |
| pectin | An01g01340 |  | GH88 | AraR | 4 | 24.79±0.04 | 0.28±0.52 | 0.22±0.01 | 0.2±0.03 | 0.1±0.02 |
| various | An07g03100 |  | CE1 |  | 4 | 128.44±3.97 | 50.94±9.77 | 39.19±4.67 | 39.26±3.73 | 39.07±3.01 |
| various | An02g02540 |  | CE16 |  | 4 | 22.68±0.68 | 3.52±0.6 | 2.16±0.8 | 1.22±0.23 | 1.3±0.09 |
| various | An07g08940 |  | CE16 |  | 4 | 10.09±0.58 | 0.07±0.15 | 0.18±0.07 | 0±0 | 0.24±0.04 |
| various | An01g12150 | lacA | GH35 | AraR, XlnR | 4 | 337.23±1.02 | 2.13±18.69 | 0.74±0.27 | 0.85±0.31 | 1.08±0.05 |
| various | An01g10350 | lacB | GH35 | AraR, GaaR | 4 | 29.46±1.27 | 8.14±0.06 | 7.06±0.54 | 5.91±0.15 | 6.43±0.32 |
| various | An09g05350 | faeE | SF9 |  | 3 | 15.25±0.01 | 29.9±0.02 | 31.86±0.5 | 45.17±0.07 | 38.83±2.63 |
|  |  |  |  |  |  |  |  |  |  |  |
